# The Neutrophil-to-Lymphocyte Ratio in Fibromyalgia: A Systematic Review with a Neuroimmune Perspective

**DOI:** 10.3390/brainsci16070703

**Published:** 2026-06-30

**Authors:** Sanam Mohammadzadeh, Shokufe Khanzade, Sarvin Es Haghi, Richard K. Shields

**Affiliations:** 1 Clinical Research Development Unit of Tabriz Valiasr Hospital, Tabriz University of Medical Sciences, Tabriz 5166614766, Iran; sanam.mhm93@gmail.com; 2 Department of Physical Therapy and Rehabilitation Science, Carver College of Medicine, The University of Iowa, 1-252 Medical Education Building (MEB), 500 Newton Rd, Iowa City, IA 52242, USA; khshokufe7@gmail.com; 3 Department of Neurology, University of Virginia, Charlottesville, VA 22903, USA; eshaghi_sarvin@yahoo.com

**Keywords:** neuroinflammation, immunity, fibromyalgia, neutrophil-to-lymphocyte ratio, NLR

## Abstract

**Highlights:**

**What are the main findings?**

- We synthesized evidence from 24 studies and demonstrated that the NLR, an index of peripheral immune balance, is greater in people with fibromyalgia.
- Our findings fit within a neuroimmune conceptual framework that emphasizes peripheral–central immune–neural interfaces and their relevance to neural adaptations in fibromyalgia.

**What are the implications of the main findings?**

- The NLR may represent a cost-effective “state-dependent” index of peripheral immune balance to help classify subgroupings of people with fibromyalgia.
- This neuroimmune framework may help inform future mechanistic studies integrating peripheral immune markers with neurobiological and clinical outcomes.

**Abstract:**

Background/Objectives. Alterations in systemic immune balance, reflected by changes in neutrophil and lymphocyte proportions, index a peripheral immune state relevant to central nervous system function. This systematic review and meta-analysis aimed to assess the differences in the neutrophil-to-lymphocyte ratio (NLR) between fibromyalgia patients and healthy controls and offer a framework to study fibromyalgia. Methods. We adhered to Preferred Reporting Items for Systematic Reviews and Meta-Analyses (PRISMA) guidelines and registered our work on PROSPERO (CRD420251001424). Two authors performed a comprehensive search of databases, including PubMed, Embase, Web of Science, and Scopus, until August 2025. The Newcastle–Ottawa Scale (NOS) was used for bias risk assessment, and STATA 19.0 software was used for statistical analyses. The standardized mean difference (SMD) and 95% confidence interval (CI) of the NLR were reported. Finally, 24 studies with 3143 fibromyalgia patients and 1610 healthy controls were included in our meta-analysis. Results. The results indicated that patients with fibromyalgia had elevated NLRs compared to healthy controls (SMD = 0.30; 95% CI = 0.07–0.52; *p* = 0.01). Our results showed no publication bias. A bivariate random-effects meta-analysis on the diagnostic accuracy of the NLR showed a summary sensitivity of 0.74 (95% CI: 0.62–0.83) and a summary specificity of 0.63 (95% CI: 0.51–0.73). Conclusions. Our meta-analysis supports the fact that the NLR is significantly different between patients with fibromyalgia and healthy controls. We conceptualize the idea that the NLR may be used as a “state-dependent” peripheral marker indexing the susceptibility of central nervous system sensitization, pain amplification, and responsiveness to future therapies.

## 1. Introduction

Fibromyalgia is a central sensitivity pain amplification disorder that has a significant social impact including absenteeism from work, lower productivity, disability and injury compensation, and the excessive use of healthcare resources [1]. Although this disorder is believed to involve mechanisms such as central sensitization, immune imbalance, neuroinflammation, and the dysfunction of the neuroendocrine and autonomic nervous systems, its precise cause is still not fully understood [2]. Increasingly, these biological mechanisms are understood within a broader biopsychosocial framework, in which immune-related and neurogenic inflammatory processes interact with psychological and clinical manifestations of fibromyalgia [3].

Numerous studies indicate that the early detection of this disease can enhance patient outcomes and allocate essential healthcare resources towards more suitable targeted therapies [4,5]. Initiatives have been undertaken to enhance diagnostic precision via revised criteria. The original 1990 American College of Rheumatology (ACR) criteria for diagnosing fibromyalgia primarily emphasized the existence of painful tender spots distributed globally [6]. Numerous modifications to the criteria have been made, with the latest revision focusing on a mix of pain and various symptoms [7]. Although diagnostic criteria for fibromyalgia have evolved over the past three decades, challenges persist, including delayed recognition, limited utility in routine practice, and frequent misdiagnoses [8]. The identification of an index that offers a broad framework for studying fibromyalgia may assist in classifying sub-types, develop mechanistic studies, and assess future therapies [1].

Recent studies have indicated the significance of neuroinflammatory pathways and biomarkers in fibromyalgia, which may serve to objectively validate a diagnosis of the condition [2]. In fact, existing evidence suggests that interactions between immune system and neural pain networks in fibromyalgia are bidirectional, occurring at both the central and peripheral levels [9]. Neutrophils and lymphocytes represent two primary components of the systemic immune balance [10]. The neutrophil-to-lymphocyte ratio has been documented as a valuable index in various pain syndromes, including chronic headache syndromes [11], trigeminal neuralgia [12], postherpetic neuralgia [13], diabetic neuropathy [14] and fibromyalgia [15,16,17,18,19,20,21,22,23,24,25,26,27,28,29,30,31,32,33,34,35,36,37,38].

Alterations in systemic immune balance, reflected by changes in neutrophil and lymphocyte proportions, may index a peripheral immune state relevant to central nervous system function [39]. Peripheral immune activity can interact with the central nervous system via cytokine-mediated signalling, autonomic pathways, and endothelial mechanisms at the blood–brain interface [39,40]. These interactions may contribute to glial priming and neuroimmune modulation, increasing susceptibility to central sensitization and pain amplification [40]. Conceptually, the NLR may represent a state-dependent peripheral index that reflects this immune–neural interface rather than a direct measure of neuroinflammation. To our knowledge, no previous meta-analysis has examined the involvement of the NLR in patients with fibromyalgia. Accordingly, we aimed to assess whether there are differences in the NLR between fibromyalgia patients and healthy controls.

## 2. Materials and Methods

### 2.1. Study Design

We adhered to Preferred Reporting Items for Systematic Reviews and Meta-Analyses (PRISMA) guidelines to maintain transparency throughout the systematic review process [41]. The completed PRISMA checklist is provided as Supplementary Tables S1 and S2. This research was registered on PROSPERO (CRD420251001424). Two authors individually carried out study selection, data extraction, and quality assessment; any disagreements were resolved by consulting a third author.

### 2.2. Search Strategy

A comprehensive search of databases, including PubMed, Embase, Scopus, and Web of Science, was performed until August 2025. The search phrases “Fibromyalgia” AND (“neutrophil to lymphocyte ratio” OR NLR) were employed to obtain pertinent articles. Medical Subject Headings (MeSH) were used in the search process. The authors also performed a comprehensive review of the reference lists of the obtained papers to identify more relevant studies. The searches were unrestricted by publication date or language. Table 1 outlines the precise search approach employed for each database.

### 2.3. Eligibility Criteria

Eligibility for inclusion in this meta-analysis was based on the following criteria: (1) Study design—observational studies. (2) Participants—patients with a definitive diagnosis of fibromyalgia and healthy controls. (3) Comparative data on blood NLR level between fibromyalgia cases and healthy controls could be obtained. The exclusion criteria included: (1) duplicate data; (2) letters, reviews, conference abstracts, or meta-analyses; (3) studies using non-human subjects. Two researchers separately conducted the preliminary screening by examining titles and abstracts and thereafter made the final determinations after evaluating the full texts. Conflicts between reviewers were discussed with a third author.

### 2.4. Data Extraction and Measurements

The information obtained from each article included the surname of the first author, publication year, sample sizes, study location, mean age, female percentage, Body Mass Index (BMI), Fibromyalgia Impact Questionnaire (FIQ) score, Visual Analogue Scale (VAS) pain score, tender point count, fibromyalgia diagnosis criteria, serum NLR in cases and controls, and NLR sensitivity and specificity in fibromyalgia.

### 2.5. Quality Assessment

The Newcastle–Ottawa Scale (NOS) was used for bias risk assessment [42], with a total score of 9 points. Studies that received scores of 0–4 were classified as low-quality, those with scores of 5–6 were deemed moderate-quality, and studies with scores from 7 to 9 were categorized as high-quality [43,44,45]. A consensus was achieved in the event of any differences during the assessment.

### 2.6. Statistical Analysis

All statistical analyses were conducted using STATA 19.0 software (StataCorp, College Station, TX, USA). The standardized mean difference (SMD) and 95% confidence interval (CI) of the NLR were computed for each included study.

Heterogeneity was evaluated using the Q test and I^2^ statistic [46]. When heterogeneity was substantial (*p* < 0.1 or I^2^ > 50%), we employed the random-effects model to assess the overall effect; otherwise, we used the fixed-effects model. Subgroup and meta-regression analyses were used to examine the probable sources of heterogeneity [47,48]. Subgroup analysis was categorized by sex, diagnostic criteria, and country. Additionally, separate univariable meta-regression analyses were performed using sample size, FIQ score, NOS score, mean age of patients, female percentage, BMI, VAS, and the number of tender point counts as covariates to investigate possible sources of heterogeneity. We used Wan et al.’s method to transform the median and interquartile range (IQR)/range to the mean and standard deviation (SD) [49].

Subgroup analyses and meta-regression were conducted using the full set of included studies to explore sources of heterogeneity. However, sensitivity analyses were performed by restricting analyses to studies reporting outcomes as the mean ± SD, thereby excluding studies requiring transformation from median-based summary statistics, to assess the robustness of the findings to data transformation.

We applied a bivariate random-effects regression model, implemented using the metandi command in STATA, to estimate sensitivity, specificity, positive and negative likelihood ratios, the diagnostic odds ratio (DOR), and their associated 95% CIs.

Begg’s rank correlation test, the creation of a funnel plot, and Egger’s linear regression were conducted to assess publication bias for the SMD meta-analysis [50]. A two-tailed *p* value of less than 0.05 was considered statistically significant.

## 3. Results

### 3.1. Search Results

**Figure 1:**
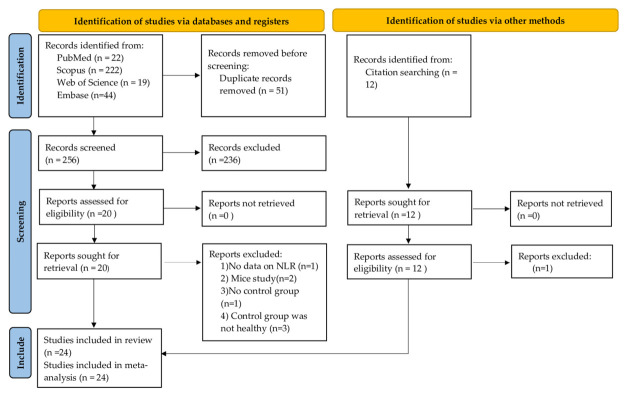
The PRISMA flow diagram of the selection process of studies.

Figure 1 shows the PRISMA flow diagram of the selection process of studies. Initially, 318 records were identified. Finally, 24 studies with 3143 fibromyalgia patients and 1610 healthy controls were included in this meta-analysis after deleting duplicates and a two-step screening of the remaining studies, the first one being based on title/abstract and the second one based on full texts [15,16,17,18,19,20,21,22,23,24,25,26,27,28,29,30,31,32,33,34,35,36,37,38]. The general characteristics of the included studies are shown in Table 2.

### 3.2. Differences in NLR Between Patients with Fibromyalgia and Healthy Controls

**Figure 2:**
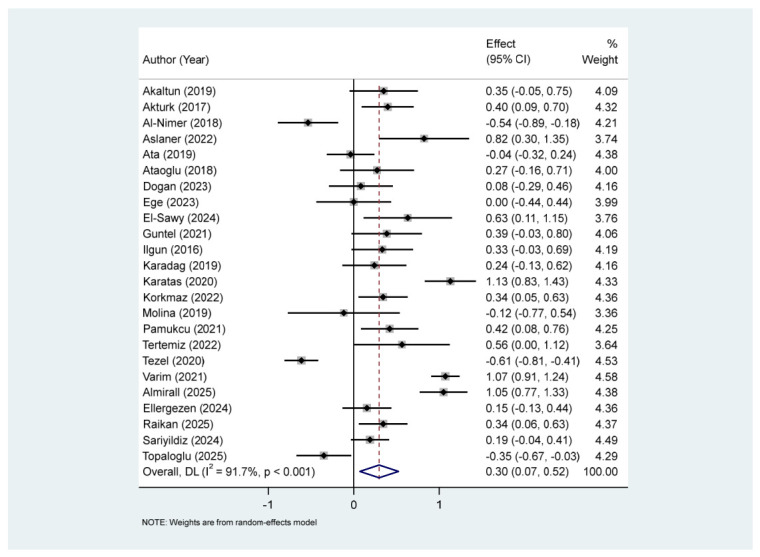
Meta-analysis of differences in NLR between patients with fibromyalgia and healthy controls.

**Figure 3:**
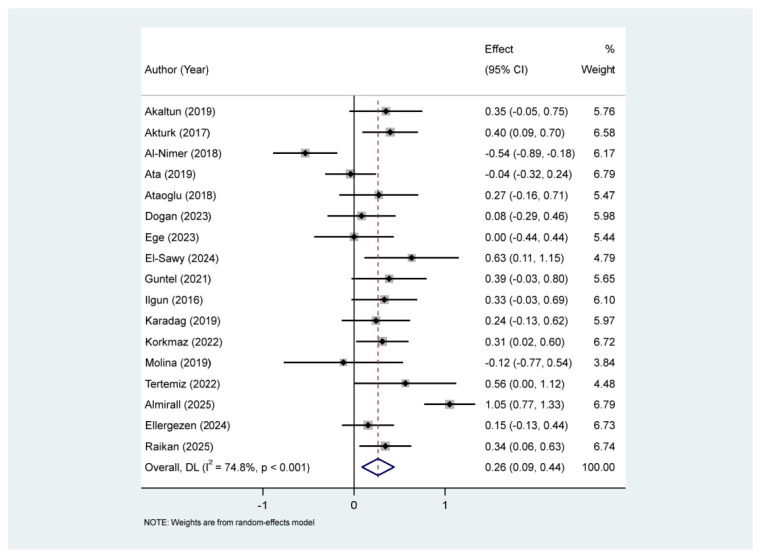
Sensitivity analysis restricted to studies reporting mean and standard deviation directly.

Given the presence of substantial heterogeneity, the pooled analysis was conducted using a random-effects model (I^2^ = 91.7%, *p* < 0.001). The results demonstrated that patients with fibromyalgia exhibited higher NLRs compared to healthy controls (SMD = 0.30; 95% CI = 0.07–0.52; *p* = 0.01), as shown in Figure 2. The sensitivity analysis restricted to studies reporting the mean and SD directly yielded comparable results (SMD = 0.26; 95% CI = 0.09–0.44; *p* = 0.003), supporting the robustness of the findings to data transformation (Figure 3). In addition, according to our predefined quality assessment criteria, one study was classified as low-quality. However, sensitivity analysis excluding this study showed that the pooled effect size remained statistically significant (SMD = 0.29; 95% CI: 0.05–0.52; *p* = 0.016), suggesting that the overall findings were robust and were not substantially influenced by its inclusion.

### 3.3. Subgroup Analysis

**Figure 4:**
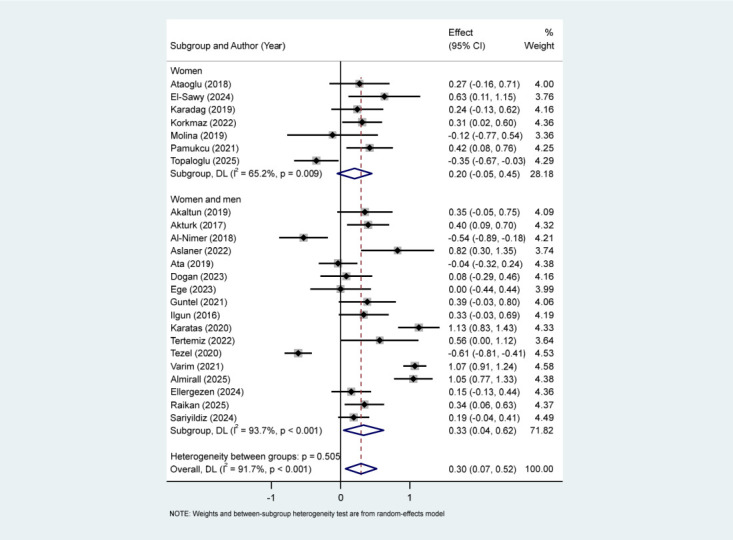
Subgroup analysis of differences in NLR between patients with fibromyalgia and healthy controls, based on sex.

When stratified by sex, studies including both women and men showed higher NLRs in fibromyalgia (SMD = 0.33; 95% CI = 0.04–0.62; *p* = 0.02), whereas studies limited to women did not show a clear difference (SMD = 0.20; 95% CI = 0.05–0.45; *p* = 0.11), as illustrated in Figure 4. The between-group comparison was not statistically significant, indicating that sex distribution did not explain heterogeneity.

**Figure 5:**
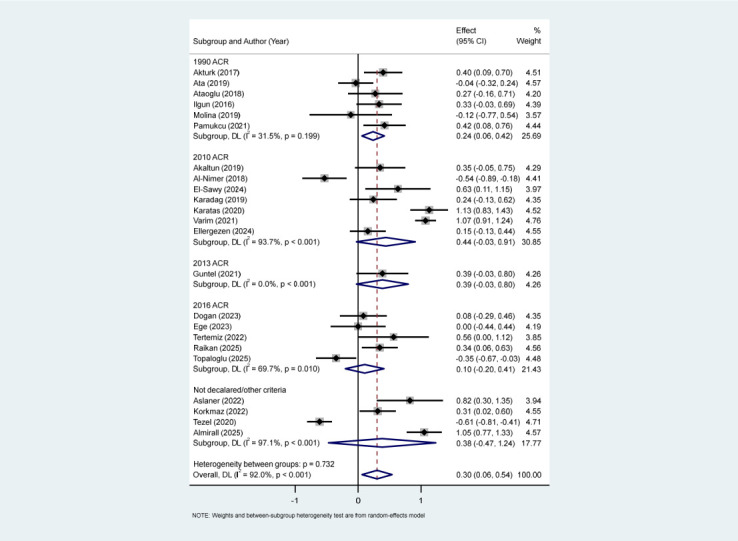
Subgroup analysis of differences in NLR between patients with fibromyalgia and healthy controls, based on diagnostic criteria.

Subgroup analysis based on diagnostic criteria showed that studies using the 1990 ACR criteria reported higher NLR values in fibromyalgia patients (SMD = 0.24; 95% CI = 0.06–0.42; *p* = 0.01). No clear difference was observed for studies using the 2010 ACR criteria (SMD = 0.44; 95% CI = −0.03–0.91; *p* = 0.06), 2013 ACR criteria (SMD = 0.39; 95% CI = −0.03–0.80; *p* = 0.06), or 2016 ACR criteria (SMD = 0.10; 95% CI = −0.20–0.41; *p* = 0.50). Similarly, studies using other or undeclared diagnostic criteria showed no clear evidence of higher NLRs (SMD = 0.38; 95% CI = −0.47–1.24; *p* = 0.38). The lack of significant between-subgroup differences indicates that diagnostic criteria did not significantly contribute to heterogeneity (Figure 5). In other words, although only studies using the 1990 ACR criteria demonstrated a statistically significant increase in the NLR, the absence of significant between-subgroup differences suggests that diagnostic criteria do not substantially modify the association between the NLR and fibromyalgia. Therefore, the observed differences across diagnostic subgroups should be interpreted cautiously and may reflect differences in statistical power rather than distinct disease phenotypes.

**Figure 6:**
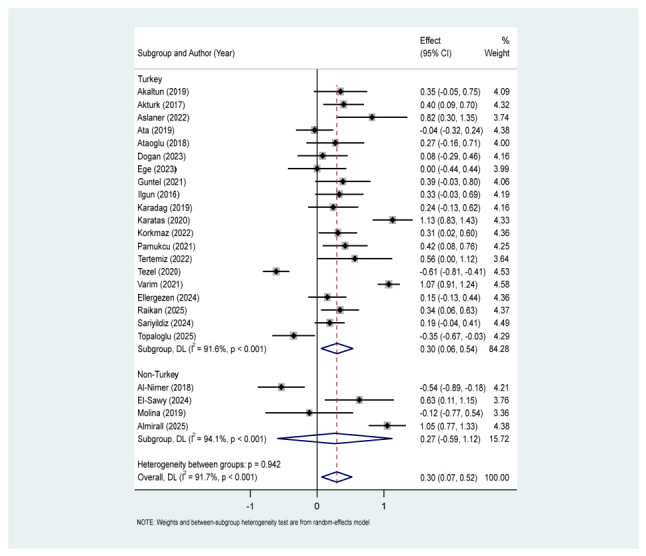
Subgroup analysis of differences in NLR between patients with fibromyalgia and healthy controls, based on country.

In addition, in the subgroup analysis based on country, we found that studies conducted in Turkey reported significantly higher NLR values in fibromyalgia patients compared with controls (SMD = 0.30; 95% CI = 0.06–0.54; *p* = 0.01). In contrast, studies conducted outside Turkey showed no significant difference in NLR values (SMD = 0.27; 95% CI = −0.59–1.12; *p* = 0.54). However, the between-subgroup heterogeneity test was not significant (*p* = 0.94), suggesting that country did not significantly contribute to the observed heterogeneity (Figure 6).

### 3.4. Meta-Regression

Sample size (coefficient = 0.0005; 95% CI: −0.0009 to 0.0020; *p* = 0.44), NOS score (coefficient = 0.006; 95% CI: −0.18 to 0.19; *p* = 0.94), BMI (coefficient = 0.04; 95% CI: −0.15 to 0.23; *p* = 0.65), mean age (coefficient = 0.01; 95% CI: −0.03 to 0.06; *p* = 0.59), female percentage (coefficient = −0.004; 95% CI: −0.04 to 0.03; *p* = 0.80), FIQ score (coefficient = 0.04; 95% CI: −0.05 to 0.13; *p* = 0.31), VAS score (coefficient = 0.16; 95% CI: −1.82 to 2.14; *p* = 0.48), and tender point count (coefficient = −0.04; 95% CI: −1.32 to 1.24; *p* = 0.97) showed no meaningful association with the effect size.

Residual heterogeneity remained high across most models (I^2^_residual = 88–93%), indicating that none of these covariates adequately accounted for the between-study variability. Although residual heterogeneity was estimated as 0% in the VAS model, this finding is limited by the inclusion of only three studies and likely reflects insufficient data rather than a true explanation of heterogeneity.

### 3.5. Sensitivity and Specificity of NLR

A bivariate random-effects meta-analysis of five studies showed a summary sensitivity of 0.74 (95% CI: 0.62–0.83) and a summary specificity of 0.63 (95% CI: 0.51–0.73). The test demonstrated a positive likelihood ratio of 1.99 (95% CI: 1.41–2.81) and a negative likelihood ratio of 0.42 (95% CI: 0.27–0.65). In addition, the DOR was 4.80 (95% CI: 2.28–10.11). The five studies included in the bivariate random-effects model for diagnostic accuracy were Aktürk et al. [15], Aslaner et al. [18], Varim et al. [37], Almirall et al. [17], and Raikan et al. [32].

### 3.6. Publication Bias

**Figure 7:**
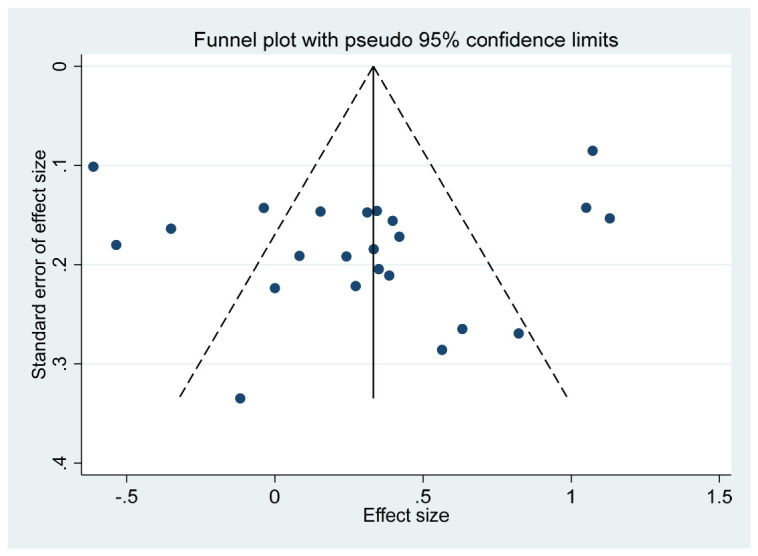
Funnel plot assessing publication bias.

As seen in our symmetrical funnel plot in Figure 7, our results showed no publication bias (Egger’s test *p* = 0.99; Begg’s test *p* = 0.70).

## 4. Discussion

Our meta-analysis showed that individuals with fibromyalgia had higher NLRs compared to healthy individuals. Although this difference was statistically significant, the effect size was small, and the specificity of the NLR was modest. These findings suggest that while the NLR may reflect underlying alterations in immune balance, its utility as a standalone diagnostic or prognostic biomarker is limited, and it should be interpreted in conjunction with clinical assessment and established diagnostic criteria. Accordingly, these findings are best interpreted as hypothesis-generating and supportive of broader mechanistic investigation rather than immediate clinical application.

The NLR illustrates the equilibrium between neutrophils, essential components of the innate immune response whose levels may be increased during acute and chronic inflammation, and lymphocytes, essential components of the adaptive immune response whose levels may be diminished in inflammatory or stress-related circumstances [10]. Recent information on this well-researched subject suggests that fibromyalgia has an immunological basis [51] that may be associated with an inflammatory state characterized by cytokines/chemokines, lipid mediators, oxidative stress, and other plasma-derived components [51]. The NLR may assist in hypothesis development for future mechanistic targets for immune and inflammatory pathways implicated in fibromyalgia.

Alongside the NLR, other biomarkers have been investigated in fibromyalgia, including genetic variations (such as polymorphisms in serotonin transporter-linked promoter region (5-HTTLPR) and Catechol-O-methyltransferase (COMT) and proinflammatory cytokines, such as interleukin (IL)-8 [1,52]. In addition, recent studies suggest that biological susceptibility in fibromyalgia may also involve genetic factors influencing psychological symptoms, stress regulation, and pain processing [53]. Incorporating genetic, epigenetic, immune, neurobiological, geographic, and secondary co-morbidity factors into the model may assist in explaining the substantial heterogeneity. In our current analysis, subgroup and meta-regression models did not identify significant contributors to the variability, and this therefore represents a key limitation when interpreting the pooled effect size. Importantly, these findings should be interpreted as primarily hypothesis-generating given the unexplained heterogeneity.

The NLR is distinguished as an economical, readily available indicator of an immunological state that can be assessed with standard blood testing [54]. Our meta-analysis highlights a clear difference between the NLR among people with and without fibromyalgia, but despite its statistical significance, the modest effect size suggests other contributing factors. Accordingly, the potential hypothesis development utility of using the NLR to stratify fibromyalgia patients based on a peripheral immune state is just one step in fully understanding the complexity of fibromyalgia. Additional research is needed to determine whether the NLR complements other cost-effective markers such as the erythrocyte sedimentation rate (ESR), C-reactive protein (CRP), genetic testing, psychological testing, and patient-reported outcomes in people with fibromyalgia.

Beyond the between-group differences demonstrated in the current meta-analysis, several individual studies have explored additional clinical applications of the NLR in fibromyalgia. Akaltun et al. reported a positive correlation between the NLR and depressive symptoms, showing that higher NLR values were associated with higher scores on the Hamilton Depression (HAM-D) rating scale (*p* < 0.05, r = 0.24) [15]. Similarly, Almirall et al. found that patients with severe disease (FIQR ≥ 59) had higher NLR values compared to those with non-severe disease (1.9 ± 0.5 vs. 1.7 ± 0.4; *p* = 0.008) [18].

Several studies have already begun to build clinical models. For example, El-Sawy et al. reported no significant correlation between the NLR and FIQR (*p* = 0.06) [24]. Likewise, Karadag et al. observed no association between the NLR and clinical indicators such as VAS or FIQ scores (*p* > 0.05) [28]. Pamukcu et al. similarly found no significant correlations between the NLR and FIQ, VAS pain, VAS fatigue, or tender point count [32]. Jayakrishnan et al. found no correlation between the NLR and FIQR or VAS pain (*p* = 0.75 and 0.44, respectively), and NLR values were comparable between patients with severe and mild/moderate VAS pain (*p* = 0.55) [55].

Other studies assessed changes in the NLR following therapeutic interventions. Inci et al. demonstrated a significant reduction in the NLR after acupuncture treatment (1.85 ± 0.09 vs. 1.56 ± 0.06, *p* < 0.001), with the NLR correlating with FIQ (*p* = 0.04) but not VAS scores (*p* = 0.12) [56]. Similarly, Aslaner et al. reported a significant decrease in the NLR following wet cupping therapy (1.80 [0.60–4.10] to 1.60 [0.49–5.87], *p* = 0.01) [19]. Taken together, the NLR illustrates the equilibrium between neutrophils, essential components of the innate immune response, and lymphocytes, essential components of the adaptive immune response, that may assist when assessing symptoms and/or interventions among people with fibromyalgia.

Fibromyalgia is increasingly conceptualized as a disorder of central nervous system pain amplification; however, the biological pathways linking peripheral physiological states to central sensitization remain incompletely understood [57]. A growing body of experimental and clinical work suggests that neuroimmune interactions, particularly those involving glial modulation, play a critical role in shaping nociceptive processing and underly fibromyalgia [58].

### 4.1. Strengths and Limitations

Our systematic review and meta-analysis possess several strengths, including a thorough evaluation of the cost-effective blood cell-derived inflammatory index, the NLR, in fibromyalgia patients; an analysis of potential correlations between effect size and predetermined study and patient characteristics; and a rigorous assessment of bias risk. This is the first meta-analysis examining the relationship between the NLR and fibromyalgia, addressing a significant void in the existing literature. We performed a comprehensive literature search across several databases—PubMed, Embase, Scopus, and Web of Science—without limitations on publication date or language, thereby ensuring the extensive coverage of available evidence. We offer a hypothesis-generating framework to inform future mechanistic studies integrating peripheral immune markers with neurobiological outcomes.

Our findings did show a heterogeneity of the pooled analysis that was not explained by our subgroup and meta-regression assessments. Our analysis supported statistically significant differences in the NLR between people with and without fibromyalgia but were unable to establish specific clinical implications associated with this effect. Future studies that are geographically widespread and that integrate peripheral immune markers such as the NLR with psychological, genetic, epigenetic, and clinical variables are needed to better understand patient heterogeneity, identify clinically meaningful subgroups, and advance personalized treatment strategies in fibromyalgia. Another limitation of our study is that the literature search was conducted through August 2025; therefore, studies published thereafter were not included.

### 4.2. Conclusions

In this systematic review and meta-analysis, we synthesized evidence from 24 observational studies and demonstrated that the NLR, an index of peripheral immune balance, is greater in fibromyalgia. However, rather than positioning the NLR as a diagnostic biomarker, we interpret our findings within a neuroimmune conceptual framework that emphasizes peripheral–central immune–neural interfaces and their potential relevance to glial priming and central sensitization. We conceptually illustrate how alterations in peripheral immune balance may interact with central nervous system processes relevant to pain amplification. This framework is explicitly hypothesis-generating and is intended to inform future mechanistic studies ultimately integrating peripheral immune markers with neurobiological, psychological, genetic, and clinical outcomes.

## Figures and Tables

**Table 1:** Exact search strategy of each database.

Scopus	ALL ((“Fibromyalgia”) AND (“Neutrophil to lymphocyte ratio” OR “NLR”))	222
Web of Science	ALL = ((“Neutrophil to lymphocyte ratio” OR “NLR”) and “Fibromyalgia”)	19
PubMed	(“Neutrophil to lymphocyte ratio” [All Fields] OR “NLR” [All Fields]) AND (“Fibromyalgia” [All Fields] OR “fibromyalgia” [MeSH Terms])	22
Embase	(“neutrophil to lymphocyte ratio”/exp OR “neutrophil to lymphocyte ratio” OR “NLR”) AND (“fibromyalgia”/exp OR “fibromyalgia”)	44

**Table 2:** Characteristics of included studies.

Author	Year	Country	Mean Age	Females %	Language	BMI	FIQ	VAS Pain	Tender Point Count	Diagnostic Criteria	Fibromyalgia Patients		Healthy Controls		Sensitivity of NLR	Specificity of NLR	NOS Score
											Number	NLR	Number	NLR			
Akaltun [15]	2019	Turkey	44.78	95.60	English	28.10	63.88	-	-	2010 ACR	91	1.96 ± 0.93	33	1.66 ± 0.6	-	-	6
Aktürk [16]	2017	Turkey	39.74	85.27	English	27.17	-	-	-	1990 ACR	197	1.93 ± 0.73	53	1.65 ± 0.6	67.5%	58%	4
Al-Nimer [17]	2018	Iraq	41.6	86.66	English	-	61.2	-	13.9	2010 ACR	150	1.99 ± 0.23	40	2.11 ± 0.20	-	-	6
Aslaner [19]	2022	Turkey	54.3	90	English	-	-	-	-	Not declared	30	1.80 [0.69–4.18]	30	1.50 [0.69–2.53]	70%	56%	5
Ata [20]	2019	Turkey	45.0	93	Turkish	26.6	-	-	-	1990 ACR	200	1.81 ± 0.67	65	1.84 ± 1.11	-	-	6
Ataoglu [21]	2018	Turkey	38.58	100	English	25.02	56.23	6.12	14.31	1990 ACR	48	2.01 ± 0.84	36	1.81 ± 0.56	-	-	7
Dogan [22]	2023	Turkey	-	-	English	-	-	-	-	2016 ACR	87	1.94 ± 0.96	40	1.87 ± 0.51	-	-	6
Ege [23]	2023	Turkey	36.9	80	English	28.8	-	-	-	ACR 2016	40	1.9 ± 0.5	40	1.9 ± 0.60	-	-	6
El-Sawy [24]	2024	Egypt	33.47	100	English	-	67.5	-	-	2010 ACR	30	1.5 ± 0.6	30	1.2 ± 0.30	-	-	8
Guntel [26]	2021	Turkey	47.23	93.0	English	-	-	-	-	2013 ACR	43	1.92 ± 0.8	49	1.66 ± 0.54	-	-	5
Ilgun [27]	2016	Turkey	43.9	80	English	28.3	-	-	15.0	1990 ACR	70	1.9 ± 0.6	52	1.7 ± 0.6	-	-	6
Karadag [28]	2019	Turkey	49.13	100	English	29.06	-	-	-	2010 ACR	98	2.07 ± 1.06	38	1.84 ± 0.59	-	-	6
Karatas [29]	2020	Turkey	48.4	-	English	-	-	-	-	2010 ACR	188	1.86 [0.13–19.83]	64	1.27 [0.59–6.11]	-	-	7
Korkmaz [30]	2022	Turkey	37.3	100	English	29.7	-	-	-	AAPT	98	2.0 ± 0.76	89	1.8 ± 0.48	-	-	7
Molina [31]	2019	Spain	52.34	100	English	26.14	60.73	6.16	-	1990 ACR	35	2.27 ± 0.99	12	2.39 ± 1.13	-	-	7
Pamukcu [32]	2021	Turkey	41.4	100	English	26.0	70.43	7.75	12.5	1990 ACR	80	1.8 [0.6–4.9]	61	1.7 [0.4–3.9]	-	-	8
Tertemiz [35]	2022	Turkey	42.7	80.76	English	-	-	-	-	2016 ACR	26	1.94 ± 0.8	25	1.54 ± 0.60	-	-	5
Tezel [36]	2020	Turkey	44.43	90.7	English	-	-	-	-	Not declared	216	1.84 [0.54–7.00]	194	1.80 [0.29–11.33]	-	-	6
Varim [38]	2021	Turkey	41.4	95.09	English	29.6	-	-	-	2010 ACR	489	3.63 (2.90–4.58)	227	2.11 (1.56–3.34)	89%	62%	6
Almirall [18]	2025	Spain	49.4	83.9%	English	27.3	71.8	-	-	Based on clinical criteria	112	1.8 ± 0.5	112	1.4 ± 0.2	70.54%	82.14%	8
Ellergezen [25]	2024	Turkey	47.84	88.99	Turkish	-	-	-	-	2010 ACR	109	1.64 ± 0.52	82	1.57 ± 0.35	-	-	9
Raikan [33]	2025	Turkey	49.10	92.66	English	-	-	-	-	2016 ACR	109	1.94 ± 0.69	85	1.71 ± 0.64	62.4%	50%	6
Sariyildiz [34]	2024	Turkey	45.17	84.5	English	-	-	-	-	ACR 1990 and/or 2010 and/or 2016	502	1.90 (1.49–2.38)	90	1.76 (1.43–2.21)	-	-	9
Topaloglu [37]	2025	Turkey	44.6	100	English	28.68	70.06	-	-	2016 ACR	95	1.83 [0.71–4.35]	63	2 [0.95–4.86]	-	-	8

Data are presented using the following formats as reported in the original studies: mean ± standard deviation (SD); median [interquartile range (IQR)]; or median (range). Bracket notation [ ] indicates interquartile ranges, whereas parentheses ( ) indicate full ranges. Mean age, mean BMI, percentage of female patients, mean FIQ, VAS pain, and tender point count refer to fibromyalgia (case) groups unless otherwise specified. NLR = Neutrophil-to-Lymphocyte Ratio; ACR = American College of Rheumatology; VAS = Visual Analogue Scale; AAPT = ACTTION-APS Pain Taxonomy; FIQ = Fibromyalgia Impact Questionnaire; NOS = Newcastle–Ottawa Scale; BMI = Body Mass Index.

## Data Availability

All data analyzed in this meta-analysis are extracted from published studies and are presented in Table 2.

## Supplementary Materials

The following supporting information can be downloaded at https://www.mdpi.com/article/10.3390/brainsci16070703/s1, Supplementary Tables S1 and S2 show completed PRISMA checklists. Table S1: PRISMA_2020_abstract_checklist. Table S2: PRISMA 2020 checklist [41].

## Abbreviations

The following abbreviations are used in this manuscript:

NLR	Neutrophil-to-lymphocyte ratio
SD	Standard deviation
IQR	Interquartile range
ACR	American College of Rheumatology
PRISMA	Preferred Reporting Items for Systematic Reviews and Meta-Analyses
FIQ	Fibromyalgia Impact Questionnaire
FIQR	Fibromyalgia Impact Questionnaire-Revised
NOS:	Newcastle–Ottawa Scale
MeSH	Medical Subject Headings
VAS	Visual analogue scale
HAM-D	Hamilton Depression
BMI	Body mass index
5-HTTLPR	Serotonin transporter-linked promoter region
COMT	Catechol-O-methyltransferase
AAPT	ACTTION-APS Pain Taxonomy
CRP	C-reactive protein
ESR	Erythrocyte sedimentation rate
DOR	Diagnostic odds ratio

## References

[1] Hackshaw K.V. (2021). The search for biomarkers in fibromyalgia. Diagnostics.

[2] García-Domínguez M. (2025). Fibromyalgia and inflammation: Unrevealing the connection. Cells.

[3] Riquelme-Aguado V., Zabarte-del Campo A., Baviano-Klett G., Fernández-Carnero J., Gil-Crujera A., Gómez-Esquer F. (2024). Correlation between different psychological variables in women with fibromyalgia with symptoms of neurogenic inflammation: A cross-sectional study. Biomedicines.

[4] Salaffi F., Farah S., Bianchi B., Lommano M.G., Di Carlo M. (2024). Delay in fibromyalgia diagnosis and its impact on the severity and outcome: A large cohort study. Clin. Exp. Rheumatol..

[5] Moshrif A., Mosallam A., Abu-Zaid M.H., Gouda W. (2023). Evaluating the effect of delayed diagnosis on disease outcome in fibromyalgia: A multi-center cross-sectional study. J. Pain Res..

[6] Wolfe F., Smythe H.A., Yunus M.B., Bennett R.M., Bombardier C., Goldenberg D.L., Tugwell P., Campbell S.M., Abeles M., Clark P. (1990). The American College of Rheumatology 1990 criteria for the classification of fibromyalgia. Arthritis Rheum. Off. J. Am. Coll. Rheumatol..

[7] Galvez-Sánchez C.M., Reyes del Paso G.A. (2020). Diagnostic criteria for fibromyalgia: Critical review and future perspectives. J. Clin. Med..

[8] Fitzcharles M.-A., Ste-Marie P.A., Pereira J.X. (2013). Fibromyalgia: Evolving concepts over the past 2 decades. Can. Med. Assoc. J..

[9] Findeisen K., Guymer E., Littlejohn G. (2025). Neuroinflammatory and immunological aspects of fibromyalgia. Brain Sci..

[10] Faria S.S., Fernandes P.C., Silva M.J., Lima V.C., Fontes W., Freitas-Junior R., Eterovic A.K., Forget P. (2016). The neutrophil-to-lymphocyte ratio: A narrative review. Ecancermedicalscience.

[11] Özdemir H.H., Dönder A. (2021). Evaluation of Neutrophil-to-lymphocyte ratio, platelet-to-lymphocyte ratio, and C-Reactive protein in tension-type headache patients. J. Neurosci. Rural. Pract..

[12] Aygün D., Doğu H. (2025). Evaluation of hematologic inflammation parameters and cranial magnetic resonance imaging findings in patients with trigeminal neuralgia. J. Health Sci. Med..

[13] Li Y., Jin J., Kang X., Feng Z. (2024). Identifying and evaluating biological markers of postherpetic neuralgia: A comprehensive review. Pain Ther..

[14] Liu S., Zheng H., Zhu X., Mao F., Zhang S., Shi H., Li Y., Lu B. (2017). Neutrophil-to-lymphocyte ratio is associated with diabetic peripheral neuropathy in type 2 diabetes patients. Diabetes Res. Clin. Pract..

[15] Akaltun M.S., Altindag O., Turan N., Aydeniz A., Gursoy S., Gur A. (2019). Can blood parameters be guiding in fibromyalgia syndrome?. Ann. Med. Res..

[16] Aktürk S., Büyükavci R. (2017). Evaluation of blood neutrophil-lymphocyte ratio and platelet distribution width as inflammatory markers in patients with fibromyalgia. Clin. Rheumatol..

[17] Al-Nimer M.S.M., Mohammad T.A.M. (2018). Correlation of hematological indices and ratios derived from them with FIQR scores in fibromyalgia. Pak. J. Med. Sci..

[18] Almirall M., Espartal E., Michelena X., Suso-Ribera C., Serrat M., Marsal S., Erra A. (2025). Neutrophil-Lymphocyte Ratio in Fibromyalgia and Axial Spondyloarthritis: A Potential Biomarker for Diagnosis and Disease Activity. Biomedicines.

[19] Aslaner H., Çaliş H., Karabaşc C., Benli A. (2022). Hemogram parameters in fibromyalgia and effects of wet cupping therapy on hemogram parameters. World J. Tradit. Chin. Med..

[20] Ata E., Düzenli T. (2019). Fibromiyalji Tanisinda Inflamatuar bir Belirteç: Platelet Dağilim Genişliği Platelet Distribution Width as a Novel Inflammatory Marker For Fibromyalgia. Bozok Tıp Derg..

[21] Ataoglu S., Ankarali H., Samanci R., Ozsahin M., Admis O. (2018). The relationship between serum leptin level and disease activity and inflammatory markers in fibromyalgia patients. North. Clin. Istanb..

[22] Dogan A. (2023). Evaluation of neutrophil-lymphocyte ratio, lymphocyte-monocyte, and monocyte-high density lipoprotein ratios in patients with fibromyalgia and determination of their relationship with disease activity, pain, and depression levels. Eur. Rev. Med. Pharmacol. Sci..

[23] Ege F., Işik R. (2023). A Comparative Assessment of the Inflammatory Markers in Patients with Fibromyalgia under Duloxetine Treatment. Front. Biosci..

[24] El-sawy E.A.M., Hakim M.M.A., El-Zohiery A.A.K., Salama S.M. (2024). Significance of Inflammatory Markers in Primary Fibromyalgia Syndrome and Their Relation in Assessing the Disease Severity. QJM Int. J. Med..

[25] Ellergezen P., Alp A., Çavun S., Çeçen G.S. (2024). Fibromiyalji Sendromunda Sistemik İmmün-İnflamasyon İndeksi ve Hematolojik Laboratuvar Bulgularına Genel Bakış. Uludağ Üniversitesi Tıp Fakültesi Derg..

[26] Guntel M., Uysal A. (2021). Detecting the presence of inflammation in fibromyalgia syndrome with neutrophil/lymphocyte ratio, platelet/lymphocyte ratio, and mean platelet volume. Ann. Med. Res..

[27] İlgün E., Akyürek Ö., Kalkan A.O., Demir F., Demirayak M., Bilgi M. (2016). Neutrophil/lymphocyte ratio and platelet/lymphocyte ratio in fibromyalgia. Eur. J. Gen. Med..

[28] Karadağ A., Hayta E. (2019). Evaluation of red blood cell distribution width, neutrophil to lymphocyte ratio and platelet to lymphocyte ratio in patients with fibromyalgia. Cumhur. Med. J..

[29] Karataş G., Gündüz R. (2020). The Significance of inflammation markers in complete blood count in patients with fibromyalgia. Med. Sci. Discov..

[30] Korkmaz M.D., Ceylan C.M. (2022). Evaluation of Inflammatory Markers in Fibromyalgia Syndrome. Med. J. Istanb. Kanuni Sultan Süleyman/İstanbul Kanuni Sultan Süleyman Tıp Derg..

[31] Molina F., Del Moral M.L., La Rubia M., Blanco S., Carmona R., Rus A. (2019). Are Patients With Fibromyalgia in a Prothrombotic State?. Biol. Res. Nurs..

[32] Pamukcu M., Baykara R.A., Duran T.I. (2021). Disease activation and laboratory parameters in Fibromyalgia Syndrome: Relationship with C-reactive protein/albumin ratio, neutrophil/lymphocyte ratio, mean platelet volume. Med. Sci..

[33] Raikan B., Ilhan K.C., Ramazan Y. (2025). Evaluation of Eosinophil-to-Lymphocyte Ratio and Eosinophil Count as Predictive Markers in Fibromyalgia Syndrome. Pak. J. Med. Sci..

[34] Sariyildiz A., Benlidayi I.C., Ornek C., Basaran S. (2024). Value of Diverse Hematological Markers in Fibromyalgia: A Real-World Study. J. Clin. Pract. Res..

[35] Tertemiz O.F., Tepe N. (2022). Is Two-Point Discrimination Test a New Diagnostic Method for the Diagnosis of Fibromyalgia?. Noropsikiyatri. Arsivi..

[36] Tezel N., Gültuna S. (2020). Comparisons of neutrophil, monocyte, eosinophil, basophil and lymphocyte ratios among the fibromyalgia syndrome and healthy individuals: Proinflammatory blood cell markers in fibromyalgia. Med. Sci. Discov..

[37] Topaloğlu M.S., Arpa M., Şen B., Bilgin Topaloğlu H., Cüre O. (2025). The Role of Immature Granulocytes in Fibromyalgia: An Indicator of Subclinical Inflammation?. Biomedicines.

[38] Varim C., Celik F., Sunu C., Kalpakci Y., Cengiz H., Öztop K., Karacer C., Yaylaci S., Gonullu E. (2021). Inflammatory Cell Ratios in the Patients with Fibromyalgia. Georgian Med. News.

[39] Prinz M., Priller J. (2017). The role of peripheral immune cells in the CNS in steady state and disease. Nat. Neurosci..

[40] Paroli M., Sirinian M.I. (2025). Pathogenic Crosstalk Between the Peripheral and Central Nervous System in Rheumatic Diseases: Emerging Evidence and Clinical Implications. Int. J. Mol. Sci..

[41] Page M.J., McKenzie J.E., Bossuyt P.M., Boutron I., Hoffmann T.C., Mulrow C.D., Shamseer L., Tetzlaff J.M., Akl E.A., Brennan S.E. (2021). The PRISMA 2020 statement: An updated guideline for reporting systematic reviews. BMJ.

[42] Wells G.A., Shea B., O’Connell D., Peterson J., Welch V., Losos M., Tugwell P. (2000). The Newcastle-Ottawa Scale (NOS) for Assessing the Quality of Nonrandomised Studies in Meta-Analyses. https://www.ohri.ca/programs/clinical_epidemiology/oxford.asp#:~:text=It%20was%20developed%20to%20assess%20the%20quality%20of,quality%20assessments%20in%20the%20interpretation%20of%20meta-analytic%20results.

[43] Dai S., Chen H., Luo T. (2023). Prevalence and factors of urinary incontinence among postpartum: Systematic review and meta-analysis. BMC Pregnancy Childbirth.

[44] Wu R., Duan M., Zong D., Li Z. (2024). Effect of arsenic on the risk of gestational diabetes mellitus: A systematic review and meta-analysis. BMC Public Health.

[45] Luchini C., Stubbs B., Solmi M., Veronese N. (2017). Assessing the quality of studies in meta-analyses: Advantages and limitations of the Newcastle Ottawa Scale. World J. Meta-Anal..

[46] Huedo-Medina T.B., Sánchez-Meca J., Marín-Martínez F., Botella J. (2006). Assessing heterogeneity in meta-analysis: Q statistic or I^2^ index?. Psychol. Methods.

[47] Baker W.L., White C.M., Cappelleri J.C., Kluger J., Coleman C.I., Health Outcomes, Policy, and Economics (HOPE) Collaborative Group (2009). Understanding heterogeneity in meta-analysis: The role of meta-regression. Int. J. Clin. Pract..

[48] Sedgwick P. (2013). Meta-analyses: Heterogeneity and subgroup analysis. BMJ.

[49] Wan X., Wang W., Liu J., Tong T. (2014). Estimating the sample mean and standard deviation from the sample size, median, range and/or interquartile range. BMC Med. Res. Methodol..

[50] Lin L., Chu H. (2018). Quantifying publication bias in meta-analysis. Biometrics.

[51] Coskun Benlidayi I. (2019). Role of inflammation in the pathogenesis and treatment of fibromyalgia. Rheumatol. Int..

[52] Ovrom E.A., Mostert K.A., Khakhkhar S., McKee D.P., Yang P., Her Y.F. (2023). A Comprehensive review of the genetic and epigenetic contributions to the development of fibromyalgia. Biomedicines.

[53] Riquelme-Aguado V., González-Álvarez M.E., Di-Bonaventura S., Rodríguez-Lagos L., Del Campo A.Z., Gómez-Esquer F., Díaz-Gil G., Gil-Crujera A. (2026). Associations Between Genetic Polymorphisms and Psychological Variables in Women With Fibromyalgia: A Cross-Sectional Study. Eur. J. Pain.

[54] Buonacera A., Stancanelli B., Colaci M., Malatino L. (2022). Neutrophil to lymphocyte ratio: An emerging marker of the relationships between the immune system and diseases. Int. J. Mol. Sci..

[55] Rajaram Jayakrishnan A.K., Easwar S.V., Thattil J., Vignesh M., Rath S., Prithvi A., Marwaha V., Cb M., Surendran S. (2022). Studying the relation between fibromyalgia severity and neutrophil-to-lymphocyte ratio, platelet-to-lymphocyte ratio, and mean platelet volume. Cureus.

[56] İnci H., İnci F. (2021). Acupuncture effects on blood parameters in patients with fibromyalgia. Med. Acupunct..

[57] Eller-Smith O.C., Nicol A.L., Christianson J.A. (2018). Potential mechanisms underlying centralized pain and emerging therapeutic interventions. Front. Cell. Neurosci..

[58] Sawicki C.M., Humeidan M.L., Sheridan J.F. (2021). Neuroimmune interactions in pain and stress: An interdisciplinary approach. Neuroscientist.

